# Developmental delay increases risk for complications within 30 days of pediatric spinal fusion surgery

**DOI:** 10.1007/s43390-025-01081-4

**Published:** 2025-04-05

**Authors:** Haseeb E. Goheer, Zachary M. Johnson, Alexander R. Garcia, Brian Q. Truong, Alden H. Newcomb, Jonathan J. Carmouche

**Affiliations:** 1 Virginia Tech Carilion School of Medicine, 2 Riverside Circle, Roanoke, VA 24016 USA; 2 Department of Orthopaedic Surgery, Carilion Clinic Institute for Orthopaedics & Neurosciences, 2331 Franklin Road Southwest, Roanoke, VA 24014 USA; 3 Edward Via College of Osteopathic Medicine, 2265 Kraft Drive, Blacksburg, VA 24060 USA; 4 Department of Orthopedics & Sports Medicine, Houston Methodist Hospital, 18123 Upper Bay Road, Houston, TX USA

**Keywords:** Developmental delay, Pediatric spinal fusion, Complications, Spinal fusion, Spine

## Abstract

**Purpose:**

The aim of this study was to investigate whether developmental delay is a risk factor for postoperative complications following pediatric spinal fusion.

**Methods:**

The American College of Surgeons National Surgical Quality Improvement Program Pediatric database was queried to retrospectively identify patients who had undergone spinal fusions between 2016 and 2021. The study population was divided into two distinct groups 1) Patients with developmental delay 2) who have no delay. T-tests for continuous variables and chi-square tests for categorical variables were used to identify differences in perioperative characteristics between the two groups. Multivariable logistic regression analysis assessed the effect of preoperative developmental delay on post-operative surgical outcomes.

**Results:**

A total of 32,621 pediatric spinal fusion patients were identified, of which 7,637 had developmental delay and 24,984 had no delay. The developmental delay group had a higher rate of surgical complications and medical complications (5.38% vs 1.41%, p < 0.001). Developmental delay independently increased the risk for medical complications (OR: 1.099, 95% CI: (1.009–1.978), surgical complications (OR: 1.4833, 95% CI (1.197–1.838), extended hospital LOS (OR: 1.250, 95% CI (1.028–1.518), intensive care unit stay (OR: 1.333, 95% CI (1.227–1.446), and death (OR: 9.638, 95% CI: 2.150–68.700) following a multivariate logistic regression analysis.

**Conclusion:**

Patients with developmental delay undergoing pediatric spinal fusion had an increased risk for surgical complications. The findings of this study serve as a valuable resource in aiding surgeons in preoperative risk assessment and in facilitating comprehensive discussions with patients and their caregivers.

## Introduction

Spinal fusion is commonly indicated in the management of scoliosis [1, 2]. 2–3% or an estimated 6–9 million people in the United States (US) are affected by scoliosis [3] Of the 2–3% of the US population with scoliosis, more than 7500 pediatric patients will undergo surgical correction of their deformity [4]. These procedures can cost up to $60,000-$180,000 per patient [5–7]. Previously published data in 2012 indicates that the costs associated with surgical correction of pediatric scoliosis are on the rise, with a national annual expenditure of $1.1 billion [6, 7]. Developmental delay is observed as a common comorbidity in pediatric patients undergoing spinal fusion [8]. Due to the heterogenous degrees of comorbidities associated with developmental delay, identifying its impact on surgical outcomes is important in risk stratifying patients and providing a more complete understanding of potential outcomes during the consent process.

Developmental delay has previously been identified as a risk factor in pediatric orthopaedic and spine procedures for postoperative complications, including infection, healthcare resource usage, and hospital readmission [9–11]. Previous investigations looking into pediatric patients undergoing treatment for open femoral shaft fractures and removal of hardware in orthopaedic procedures found developmental delay to be independently associated with increased risk of hospital readmission and increased risk of infection [10, 11]. Pediatric patients with developmental delay may present with additional challenges in relation to the complexity of their care such as requiring increased physician visits, emergency department visits, hospitalizations, and visits to other healthcare practitioners [12]. Although previous spine surgery research has shown the negative impact of intellectual disability and Down syndrome on surgical outcomes in pediatric spinal fusion, limited studies have investigated the connection between developmental delay and outcomes in pediatric spinal fusion procedures. A retrospective analysis of 23 pediatric patients with Down syndrome undergoing pediatric spinal fusion found that 52% experienced at least one postoperative complication, a higher rate than in children without developmental delay undergoing the same procedure ranging from 5 to 23% [9, 13–15]. The studies investigating how developmental delay impact pediatric spinal fusion outcomes have been limited by their smaller sample size or have been conducted at a single institution with non-representative samples of the national population [1, 9]. The purpose of our study is to compare demographic and comorbidity profiles with postoperative outcomes in children with and without developmental delay undergoing pediatric spinal fusion using a large multi-institutional database. To our knowledge, no studies have specifically evaluated developmental delay as an independent risk factor for unfavorable outcomes following pediatric spinal fusion. These findings will improve risk stratification for patients with developmental delay undergoing complex spinal fusion procedures.

## Materials and methods

### Study design and data sources

This is a retrospective study of patients in the American College of Surgeons National Surgical Quality Improvement Program Pediatric (ACS-NSQIP- Pediatric) database. The ACS-NSQIP-Pediatric is a national surgical database of data from hospitals providing pediatric care throughout the United States. The participating institutions have certified clinical reviewers responsible for verifying the authenticity of the data and the database has regular audits [16]. IRB approval was not requested as the database contains de-identified information.

### Disclaimer

*American College of Surgeons National Surgical Quality Improvement Program and the hospitals participating in the ACS NSQIP are the source of the data used herein; they have not verified and are not responsible for the statistical validity of the data analysis or the conclusions derived by the authors.*

### Study population

**Fig. 1:**
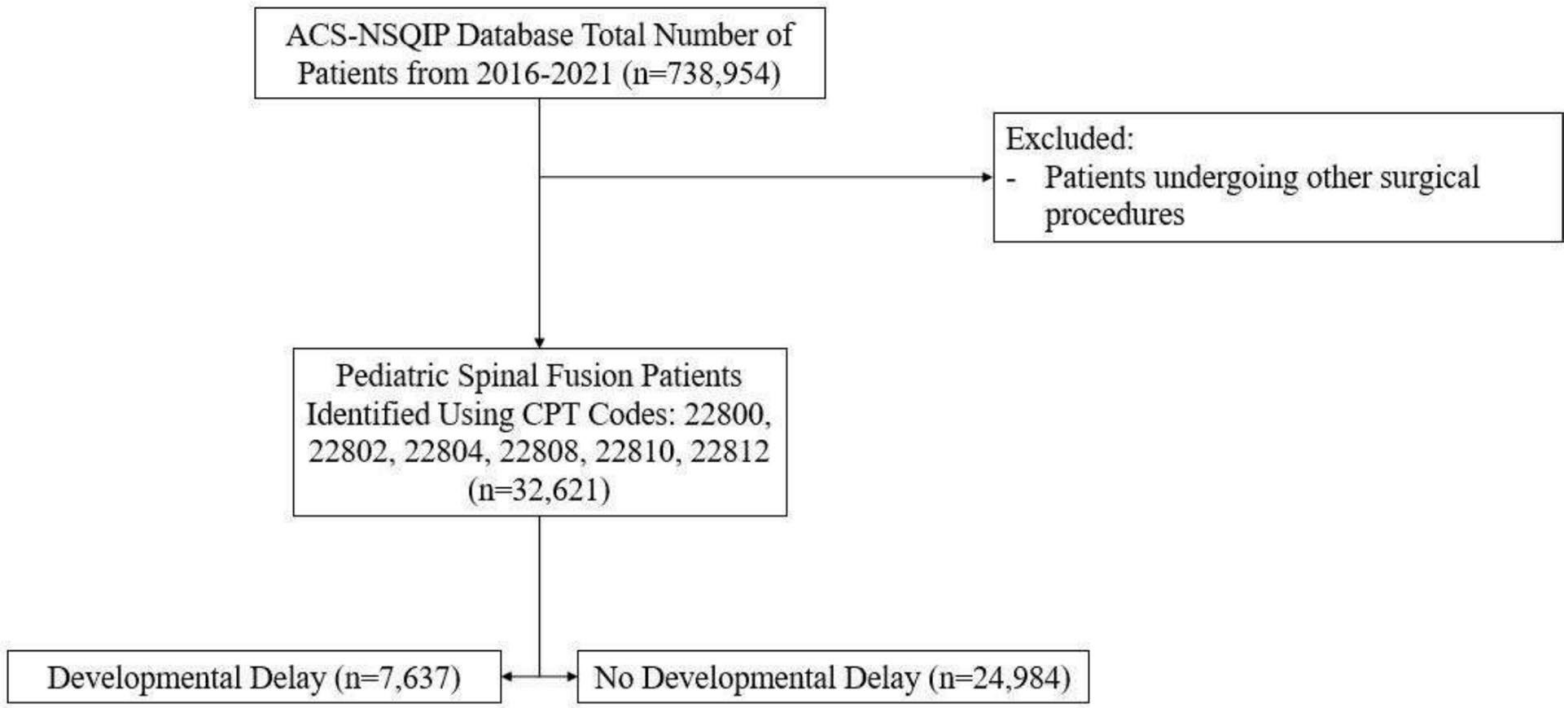
Flow diagram of the cohort selection process

Data from 2016 to 2021 were reviewed to collect data on patients undergoing pediatric spinal fusion procedures. Patients were identified using the Current Procedural Terminology codes 22800, 22802, 22804, 22808, 22810, 22812. The identified and exclusions are outlined in the CONSORT flow chart (Fig. 1). Using unique case identifiers, the ACS-NSQIP Pediatric dataset was merged with the ACS-NSQIP Pediatric Spinal Fusion Procedure Targeted dataset. Patients not found in both datasets were excluded from the study. Patients identified in the database to have a developmental delay were assigned to the developmental delay cohort while all remaining patients were assigned to the no developmental delay cohort. Developmental delay, defined by impaired cognitive status, was a predefined variable in the ACS-NSQIP database. The clinical diagnosis of developmental delay was determined by certified clinical reviewers after electronic medical record review.

### Patient variables and outcomes of interest

Patient and clinical characteristics collected from the ACS-NSQIP-Pediatric database included demographic variables, medical comorbidities, intraoperative data, and postoperative outcomes within the first 30 postoperative days. The list of preoperative comorbidity variables includes: ventilator requirement, asthma, history of chronic lung disease (CLD), tracheostomy, esophageal or intestinal disease, prior cardiac surgery, cardiac risk factors, pulmonary abnormalities, impaired cognition status, seizure, intraventricular hemorrhage, steroid use within 30 days of surgery, nutritional support, bleeding disorder, hematological disorder, previous sepsis, childhood malignancy, and the American Society of Anesthesiologist (ASA) classification. Outcomes were categorized by the presence or absence of medical and surgical complications. Surgical complications included wound disruption, superficial surgical site infection (SSI), deep SSI, organ SSI, or wound dehiscence. Medical complications included pneumonia, unplanned intubation, renal insufficiency, acute renal failure, urinary tract infection (UTI), stroke with neurological deficit, seizure, nerve injury, cardiac arrest, bleeding transfusion, vein thrombosis, sepsis, *Clostridioides difficile* colitis, reoperation, extended length of stay or intensive care unit (ICU) stay. Extended hospital length of stay (LOS) was defined by a stay greater than or equal to two standard deviations above the mean.

### Statistical analysis

Differences in pre- and peri-operative variables were compared between the developmental delay cohorts using t-test for continuous variables and chi-square tests for categorical variables. Multivariate logistic regression analyses were used to determine independent associations of developmental delay with the occurrence of medical and surgical complications, intensive care unit stay, and extended length of stay, within thirty days. We included preoperative variables with n > 10 and *p* < 0.05 from the covariate screening in the multivariate logistic regression. Given the large number of potential confounders, multivariate analyses were completed using model selection by Akaike information criterion in backward stepwise regression to isolate development delay as a risk factor while controlling for pre-operative comorbidities and demographic characteristics. Significant variables initially in the regression included age, sex, race, admission status, surgical approach, scoliosis type, ventilator dependence, asthma, chronic lung disease, oxygen support, tracheostomy, esophageal, gastric or intestinal disease, history of cardiac surgery, cardiac risk factors, pulmonary abnormalities, seizure, cerebral palsy, structural CNS abnormalities, neuromuscular disorder, intraventricular hemorrhage, steroid use, ostomy, pre-operative wound infection, nutritional support, sepsis prior to surgery, hematologic disorders, cardiovascular pulmonary resuscitation within seven days of surgery, transfusions, childhood malignancy, ASA class, type of fusion, and operative time. Significance was defined as *p* < 0.05. All statistical analyses were performed using the statistical coding program R version 4.3.3 (R, Vienna, Austria).

## Results

### Developmental delay and the overall pediatric spinal fusion population

A total of 32,621 pediatric spinal fusion patients were identified, of which 7,637 (23.4%) had developmental delay (Table 1). The developmental delay cohort was significantly younger (*p* < 0.001) and contained significantly more males compared to the control population (*p* < 0.001). Significant differences in comorbidities between the two groups were identified. Compared to the no developmental delay group, the developmental delay group had higher rates of most comorbidities with statistically significant associations. This list includes ventilator dependence (7.95%; *p* < 0.001), asthma (16.6%; *p* < 0.001), CLD (16.3%; *p* < 0.001), oxygen support (6.5%; *p* < 0.001), tracheostomy (6.8%; *p* < 0.001), history of cardiac surgery (9.9%; *p* < 0.001), severe cardiac risk factors (0.9%; *p* < 0.001), pulmonary abnormalities (20.8%; *p* < 0.001), seizure (40.9%; *p* < 0.001), cerebral palsy (38.5%; *p* < 0.001), structural CNS abnormalities (37.3%; *p* < 0.001), neuromuscular disorder (63.7%; *p* < 0.001), intraventricular hemorrhage (3.3%), steroid use (2.4%; *p* < 0.001), wound infection (1.0%; *p* < 0.001), nutritional support (33.2%; *p* < 0.001), hematologic disorders (6.0%; *p* < 0.001), childhood malignancy (1.5%; *p* = 0.031), and an ASA classification greater or equal to 3 (80.6%; *p* < 0.001).

**Table 1:** Demographics variables and comorbidities in patients undergoing pediatric spinal fusion (N = 32,621)

	Developmental Delay n = 7,637	No Delay n = 24,984	*p*
Age	12.28 (3.16)	13.61 (2.45)	**< 0.001**
Sex			
Female	53.14	72.91%	**< 0.001**
Male	46.86	27.09%	
Race	65.54%	64.81%	0.252
White	15.14%	15.37%	
Black	2.78%	3.32%	
Asian	0.55%	0.37%	
American Indian or Alaskan Native	0.37%	0.23%	
Native Hawaiian or Pacific Islander	15.63%	15.90%	
Unknown	65.54%	64.81%	
Admission status			
Inpatient	97.89%	97.89%	0.519
Outpatient	2.11%	2.11%	
Surgical approach			
Anterior	2.15%	2.85%	**< 0.001**
Posterior	97.85%	97.15%	
Scoliosis			
Congenital/Structural	6.77%	6.66%	**< 0.001**
Idiopathic	16.57%	78.69%	
Unable to classify	1.34%	1.32%	
Kyphosis	4.79%	4.48%	
Neuromuscular	62.34%	6.42%	
Syndromic	8.19%	2.41%	
Ventilator dependence	7.95%	0.86%	**< 0.001**
Asthma	16.62%	5.23%	**< 0.001**
Chronic lung disease	16.34%	1.86%	**< 0.001**
Oxygen support	6.53%	0.37%	**< 0.001**
Tracheostomy	6.81%	0.44%	**< 0.001**
Esophageal, Gastric, or intestinal disease	30.2%	3.55%	**< 0.001**
History of cardiac Surgery	9.86%	2.39%	**< 0.001**
Cardiac risk factors			
Severe cardiac risk factors	0.90%	0.34%	**< 0.001**
Major cardiac risk factors	9.31%	2.69%	
Minor cardiac risk factors	11.18%	3.11%	
No cardiac risk factors	78.60%	93.85%	
Pulmonary Abnormalities	20.81%	2.62%	**< 0.001**
Seizure	40.85%	1.38%	**< 0.001**
Cerebral palsy	38.47%	1.14%	**< 0.001**
Structural CNS abnormalities	37.37%	6.4%	**< 0.001**
Neuromuscular Disorder	63.69%	8.12%	**< 0.001**
Intraventricular Hemorrhage	3.26%	0.16%	**< 0.001**
Steroid use	2.37%	0.55%	**< 0.001**
ostomy	40.41%	1.77%	**< 0.001**
Wound infection	0.98%	0.16%	**< 0.001**
Nutritional support	33.17%	1.07%	**< 0.001**
Sepsis prior to surgery	0.92%	0.51%	**< 0.001**
Hematologic disorders	6.06%	1.84%	**< 0.001**
Inotropic support at Time of surgery	0.52%	0.49%	0.804
CPR within 7 days of Surgery	0.08%	0.02%	**0.018**
Transfusions	0.33%	0.14%	**< 0.001**
Childhood malignancy	1.49%	0.97%	**< 0.001**
ASA Class			
I: No disturbance	1.00%	21.01%	**< 0.001**
II: Mild disturbance	18.16%	60.15%	
III: Severe disturbance	73.10%	17.72%	
IV: Life threatening	7.45%	0.94%	
V: Moribund	0.03%	0.00%	
Work RVU (mean)	33.64	32.34	**< 0.001**
Laparoscopic/Minimally Invasive surgery	1.55%	1.69%	0.417
Type of fusion			
Primary	84.46%	92.30%	**< 0.001**
Revision	15.54%	7.70%	
Operative time (minutes)	315.56 (119.21)	277.78 (102.47)	**< 0.001**

*ASA* American society of anesthesiologists, *RVU* Relative value units

### Developmental delay and pediatric spinal fusion procedure characteristics

Overall, patients with developmental delay experienced longer operative times (315.6 min vs 277.8 min; p < 0.001) and a higher prevalence of procedures using a posterior surgical approach (97.9% vs 97.2%; p < 0.001) (Table 1). Patients without developmental delay had a higher prevalence of patients classified as neuromuscular scoliosis (62.3% vs 6.4%) compared to patients with no developmental delay who had a greater proportion of scoliosis classified as idiopathic (78.7% vs 16.6%; p < 0.001). Patients with developmental delay had a higher intraoperative use of antifibrinolytic (89.7% vs 88.0%; p < 0.001) and postoperative ICU stay (6.2% vs 1.6%; p < 0.001) (Table 3) while patients with no developmental delay had a higher proportion of patients who had a pre-operative MRI for spinal fusion (38.4% vs 25.0%; p < 0.001) (Table 2).

**Table 2:** Univariate analysis of pediatric spinal fusion characteristics by developmental delay cohort

	Developmental Delay n = 7,637	No Delay n = 24,984	*p*
Pre-operative MRI for spinal fusion	24.97%	38.42%	**< 0.001**
Intensive care unit stay	6.22%	1.64%	**< 0.001**
Intraoperative use of neuromonitoring	93.57%	96.89%	**< 0.001**
Intraoperative use of antibiotics	84.92%	81.54%	**< 0.001**
Intraoperative use of antifibrinolytics	89.69%	87.98%	**< 0.001**
Postoperative neurological deficit	1.11%	1.3%	0.218

Values are bolded for *p* < 0.05

### Postoperative complications and multivariate analyses

Patients with developmental delay had a higher rate of having the occurrence of one or more surgical complications or medical complications (5.4% vs 1.4%; p < 0.001) compared to patients with no developmental delay. The prevalence of post-operative pneumonia (3.3% vs 0.3%; p < 0.001), reoperation (21.4% vs 7.2%; p < 0.001), and extended length of stay (6.2% vs 1.6%; p < 0.001) was greater in cohort with developmental delay (Table 3). Multivariate logistic regression was performed to determine the differential effect of developmental delay compared to the no developmental delay group on medical complications, surgical complications, ICU stay, extended length of stay, and death while adjusting for the greater comorbidity burden (Table 4). Patients with developmental delay were found to have an increased risk of (OR: 1.099, 95% CI (1.009–1.978), surgical complications (OR: 1.4833, 95% CI (1.197–1.838), extended hospital LOS (OR: 1.250, 95% CI (1.028–1.518), intensive care unit stay (OR: 1.333, 95% CI (1.227–1.446), and death (OR: 9.638, 95% CI 2.150–68.700).

**Table 3:** Univariate analysis of thirty-day post-operative complications after pediatric spinal fusion by cohort

	Developmental Delay n = 7,637	No Delay n = 24,984	*p*
Death	0.32%	0.01%	**< 0.001**
surgical complications	5.38%	1.41%	**< 0.001**
Superficial incisional surgical site infection	1.78%	0.62%	**< 0.001**
Deep incisional surgical site infection	2.11%	0.41%	**< 0.001**
Organ space surgical site infection	0.9%	0.19%	**< 0.001**
Wound disruption	1.24%	0.35%	**< 0.001**
Medical Complications	5.38%	1.41%	**< 0.001**
Pneumonia	3.31%	0.29%	**< 0.001**
Unplanned intubation	2.12%	0.13%	**< 0.001**
Renal insufficiency	0.09%	0.04%	0.104
Acute renal failure	0.07%	0.01%	**0.011**
Urinary tract infection (UTI)	1.95%	0.22%	**< 0.001**
Coma > 24 hours	0.03%	0.00%	0.085
Stroke or intracranial hemorrhage	0.03%	0.01%	0.728
Seizure	0.16%	0.03%	**< 0.001**
Nerve injury	0.22%	0.37%	0.063
Cardiac arrest Requiring CPR	0.42%	0.03%	**< 0.001**
Bleeding/transfusions	76.08%	66.9%	**< 0.001**
deep vein Thrombosis	0.45%	0.07%	**< 0.001**
C. diff Colitis	0.62%	0.06%	**< 0.001**
Sepsis	2.07%	0.21%	**< 0.001**
Reoperation	21.37%	7.18%	**< 0.001**
Extended length of hospital stay	6.22%	1.64%	**< 0.001**

Values are bolded for *p* < 0.05

**Table 4:** Multivariate adjusted 30-day pediatric spinal fusion postoperative outcomes between groups

Overall Cohort	Developmental Delay (n = 7,637)			*p*
	Adjusted Odds Ratio*	95% CI		
		Lower	Upper	
Medical complications	1.099	1.009	1.978	**0.032**
surgical Complications	1.619	1.230	2.131	** < 0.001**
extended hospital LOS	1.250	1.028	1.518	**0.025**
Intensive care unit stay	1.400	1.292	1.517	** < 0.001**
Death	9.638	2.150	68.700	**0.007**

Values are bolded for *p* < 0.05

^*^Relative to No Delay cohort

## Discussion

This study represents the first large-scale database study evaluating associations between demographic and comorbidity profiles in patients with developmental delay and post-operative outcomes in patients undergoing pediatric spinal fusion surgery. The most important finding of this study was the finding that developmental delay was an independent predictor for medical complications, surgical complications, extended hospital LOS, ICU stay, and death in pediatric spinal fusion patients. As the available research showing associations between developmental delay and poor health and surgical outcomes grows, it is important to understand how this information affects pediatric spinal fusion outcomes.

Several studies have previously elucidated poor surgical outcomes among pediatric patients with developmental delay [9–11]. Dobek et al. described a population of pediatric femoral shaft fracture patients with and without developmental delay. They found that developmental delay was an independent risk factor for hospital readmission, concluding that individual treatment plans should be created to optimize readmission risk and lower costs for the patient and hospital [11]. Boulos et al. evaluated pediatric patients undergoing open reduction internal fixation for upper and lower extremity fracture as well as those undergoing hardware removal due to hardware complications [10]. They discovered that patients who underwent hardware removal were more likely to suffer from developmental delay. Developmental delay subsequently was defined as a key risk factor for infection-relation removal and subsequent investigation was recommended [10]. Fraser et al. conducted a retrospective analysis of patients with Down syndrome treated with spinal fusion. Their analysis showed that the high prevalence of medical comorbidities suggests that the population with Down Syndrome is medically complex and requires meticulous preoperative and postoperative individualized care [9].

The challenges of treating these patients extend beyond comorbidities seen within the population. Despite investigating psychosocial factors affecting treatment of pediatric patients with developmental delay, our analysis demonstrates the challenge of treatment extends beyond the comorbidities seen in this population [17, 18]. These patients can have difficulty following postoperative care instruction and expressing pain, resulting in increased agitation necessitating additional psychosocial support in their recovery [17, 19]. The importance of psychosocial factors in the care of all pediatric patients has been highlighted by Richard et al. in their investigation of pediatric patients undergoing external fixation, and this can likely be extended to developmental delay patients and the increased complications seen in this population [17]. Additionally, Chun et al. postulated that factors such as ability to communicate, tolerance of postoperative eye drops and examinations, and self-injurious behaviors contributed to the increased association endophthalmitis in their investigation of pediatric cataract surgery complications [20]. These previous investigations in addition to our multivariate analysis highlight the importance of preoperative and postoperative care needs in this specific population.

Our study also demonstrates that despite undergoing significantly more preoperative preparation and optimization, shown by increased preoperative MRI, ICU stay, intraoperative neuromonitoring, and antifibrinolytic usage, patients with developmental delay still experienced more surgical complications when controlling for demographic differences. This finding emphasizes the need for preoperative discussions with patients and risk assessment. This population also requires in-depth behavioral and stress-reduction strategies to facilitate all stages of the perioperative experience. Patients with developmental delay can have difficulties with communication and social interaction which can make the preoperative and perioperative environment difficult [21]. Understanding these factors will help optimize care for pediatric patients with developmental delay receiving spinal fusion.

This study has several limitations. The retrospective design and use of the ACS-NSQIP database limits our ability to randomize our patient population and control specific variables, such as variations in postoperative intensive care unit stay protocols. Additionally, developmental delay is a predetermined variable available within the database. The variable is captured by certified clinical reviewers. Although ACS-NSQIP database collects data on a wide array of predetermined factors, it does not include patient-reported outcomes or radiological results. Furthermore, the database only captures information up to thirty days after surgery. This limits our capacity to assess the long-term outcomes, such as fusion data, and may result in the exclusion of unreported complications (e.g. pseudoarthrosis and hardware failure) and functional outcomes beyond the thirty-day period. Evaluating these complications is crucial for assessing the overall efficacy and potential risks associated with pediatric spine surgery. Although the database captures a wide variety of complications; a reduced number of occurrences may contribute to large confidence intervals [22]. Additionally, the study design does not allow us to establish a causal relationship between developmental delay and postoperative complications. Despite these limitations, we were able to find an association between developmental delay in pediatric spinal fusion patients and increased chances of surgical complications and ICU stay. This information can aid surgeons in identifying patients in need of not only medical optimization, but more personalized care due to differences in communication in this population.

In conclusion, patients with developmental delay undergoing pediatric spinal fusion had an increased risk for surgical complications. The findings of this study serve as a valuable resource in aiding surgeons in preoperative risk assessment and in facilitating comprehensive discussions with patients and their caregivers. Given the heterogenous nature presentation of developmental delay within the pediatric population, personalized postoperative management, guided by risk assessment, may help mitigate postoperative complications.

## Data Availability

The data that support the findings of this study are available from ACS-NSQIP. Restrictions apply to the availability of these data, which were used under a data use agreement for this study.

## References

[1] Ghandhari H, Ameri E, Nikouei F et al (2018) Long-term outcome of posterior spinal fusion for the correction of adolescent idiopathic scoliosis. Scoliosis Spinal Disord 13:1–5 30123840 10.1186/s13013-018-0157-z PMC6090875

[2] Loughenbury PR, Tsirikos AI (2022) Current concepts in the treatment of neuromuscular scoliosis: clinical assessment, treatment options, and surgical outcomes. Bone Jt Open 3:85–92 35084206 10.1302/2633-1462.31.BJO-2021-0178.R1 PMC9047085

[3] Scoliosis. AANS. Published April 30, 2024. https://www.aans.org/patients/conditions-treatments/scoliosis/#:~:text=Scoliosis%20affects%202%2D3%20percent.

[4] George J, Das S, Egger AC et al (2019) Influence of intraoperative neuromonitoring on the outcomes of surgeries for pediatric scoliosis in the United States. Spine Deform 7(1):27–32 30587317 10.1016/j.jspd.2018.05.013

[5] Barsdorf AI, Sproule DM, Kaufmann P (2010) Scoliosis surgery in children with neuromuscular disease: findings from the US National Inpatient Sample, 1997 to 2003. Arch Neurol 67(2):231–235 20142532 10.1001/archneurol.2009.296

[6] Vigneswaran HT, Grabel ZJ, Eberson CP et al (2015) Surgical treatment of adolescent idiopathic scoliosis in the United States from 1997 to 2012: an analysis of 20,346 patients. J Neurosurg Pediatr 16(3):322–328 26114991 10.3171/2015.3.PEDS14649

[7] Martin CT, Pugely AJ, Gao Y et al (2014) Increasing hospital charges for adolescent idiopathic scoliosis in the United States. Spine 39(20):1676–1682 24983937 10.1097/BRS.0000000000000501

[8] AlNouri M, Wada K, Kumagai G et al (2023) Diseases and comorbidities associated with early-onset scoliosis: a retrospective multicenter analysis. Spine Deform 11:481–486 36380109 10.1007/s43390-022-00613-6

[9] Fraser HG, Krakow A, Lin A et al (2022) Outcomes of posterior spinal fusion in pediatric patients with down syndrome. J Bone Joint Surg 104:2068–2073 36166508 10.2106/JBJS.22.00588

[10] Boulos A, DeFroda SF, Kleiner JE et al (2017) Inpatient orthopaedic hardware removal in children: a cross-sectional study. J Clin Orthop Trauma 8:270–275 28951646 10.1016/j.jcot.2017.06.020 PMC5605744

[11] Dobek A, Quan T, Manzi JE et al (2023) Developmental delay: is this pediatric patient population at risk for complications following open treatment of femoral shaft fracture? Eur J Orthop Surg Traumatol 33:1751–1756 35945391 10.1007/s00590-022-03348-2

[12] Gallaher MM, Christakis DA, Connell FA (2002) Health care use by children diagnosed as having developmental delay. Arch Pediatr Adolesc Med 156:246–251 11876668 10.1001/archpedi.156.3.246

[13] Weiss HR, Goodall D (2008) Rate of complications in scoliosis surgery - a systematic review of the Pub Med literature. Scoliosis. 10.1186/1748-7161-3-9 18681956 10.1186/1748-7161-3-9 PMC2525632

[14] Murphy RF, Mooney JF 3rd (2016) Complications following spine fusion for adolescent idiopathic scoliosis. Curr Rev Musculoskelet Med 9(4):462–469. 10.1007/s12178-016-9372-5 27639726 10.1007/s12178-016-9372-5 PMC5127952

[15] Roberts SB, Tsirikos AI (2022) Paediatric Spinal Deformity Surgery: Complications and Their Management. Healthcare (Basel). 10.3390/healthcare10122519 36554043 10.3390/healthcare10122519 PMC9778654

[16] Shiloach M, Frencher SK Jr, Steeger JE et al (2010) Toward robust information: data quality and inter-rater reliability in the American college of surgeons national surgical quality improvement program. J Am Coll Surg 210(1):6–16. 10.1016/j.jamcollsurg.2009.09.031 20123325 10.1016/j.jamcollsurg.2009.09.031

[17] Richard HM, Nguyen DC, Birch JG, Roland SD, Samchukov MK, Cherkashin AM (2015) Clinical implications of psychosocial factors on pediatric external fixation treatment and recommendations. Clin Orthop Relat Res 473(10):3154–3162. 10.1007/s11999-015-4276-z 25828943 10.1007/s11999-015-4276-z PMC4562937

[18] Maloy GC, Kaszuba SV, Stoeckel M, Mariotti EC, Frumberg DB (2023) A practical guide for improving orthopaedic care in children with autism spectrum disorder. J Pediatric Orthopaedic Soc North Am. 10.55275/jposna-2023-640 10.55275/JPOSNA-2023-640 PMC12088194 40433087

[19] Vialle R, Thévenin-Lemoine C, Mary P (2013) Neuromuscular scoliosis. Orthop Traumatol Surg Res 99(1):S124–S139. 10.1016/j.otsr.2012.11.002 23337438 10.1016/j.otsr.2012.11.002

[20] Chun LY, Tanenbaum RE, Liao C, Rodriguez SH (2023) The association between developmental delay and endophthalmitis after pediatric cataract surgery using an insurance claims database. J AAPOS 27(6):331.e1-331.e6. 10.1016/j.jaapos.2023.09.003 39195355 10.1016/j.jaapos.2023.09.003

[21] Section on Anesthesiology and Pain Medicine (2014) The pediatrician’s role in the evaluation and preparation of pediatric patients undergoing anesthesia. Pediatrics 134(3):634–641 25157004 10.1542/peds.2014-1840

[22] Dorey FJ (2010) In brief: statistics in brief: Confidence intervals: what is the real result in the target population? Clin Orthop Relat Res 468(11):3137–3138. 10.1007/s11999-010-1407-4 20532716 10.1007/s11999-010-1407-4 PMC2947664

